# Lentinan from shiitake selectively attenuates AIM2 and non-canonical inflammasome activation while inducing pro-inflammatory cytokine production

**DOI:** 10.1038/s41598-017-01462-4

**Published:** 2017-05-02

**Authors:** Huijeong Ahn, Eunsaem Jeon, Jin-Chul Kim, Seung Goo Kang, Sung-il Yoon, Hyun-Jeong Ko, Pyeung-Hyeun Kim, Geun-Shik Lee

**Affiliations:** 1 Laboratory of Physiology and Inflammatory Diseases, College of Veterinary Medicine and Institute of Veterinary Science, Kangwon National University, Chuncheon, 24341 Republic of Korea; 2 Natural Products Research Center, Korea Institute of Science and Technology, Gangneung, 25451 Republic of Korea; 3 Department of Molecular Bioscience, School of Biomedical Science, Kangwon National University, Chuncheon, 24341 Republic of Korea; 4 Division of Biomedical Convergence, College of Biomedical Science, Kangwon National University, Chuncheon, 24341 Republic of Korea; 5 Laboratory of Microbiology and Immunology, College of Pharmacy, Kangwon National University, Chuncheon, 24341 Republic of Korea

## Abstract

Lentinan extracted from shiitake (*Lentinula edodes*) is a β-glucan that has been reported as an intravenous anti-tumor polysaccharide via enhancement of the host immune system. In this study, we determined the effect of lentinan on inflammasome activation, a multi-protein platform, in myeloid cells. Mouse bone marrow-derived macrophages were treated with lentinan with/without inflammasome triggers, and maturation of interleukin (IL)-1β, IL-18, or caspase-1 was measured as a readout of inflammasome activation. As a result, lentinan selectively inhibited absent in melanoma 2 (AIM2) inflammasome activation. In addition, lentinan up-regulated pro-inflammatory cytokines and induced expression of inflammasome-related genes through toll-like receptor 4 signaling. Furthermore, we assessed the effect of lentinan on mice treated with *Listeria monocytogenes* or lipopolysaccharide as an AIM2 or non-canonical inflammasome-mediated model. Lentinan attenuated IL-1β secretion resulting from *Listeria-*mediated AIM2 inflammasome activation and reduced endotoxin lethality via inhibition of non-canonical inflammasome activation. Thus, lentinan is suggested as an anti-AIM2 and anti-non-canonical inflammasome candidate despite its up-regulation of cytokine expression.

## Introduction

β-glucans, which have a long history as non-specific immunomodulators, are heterogeneous polysaccharides of glucose polymers^1, 2^. β-glucans can enhance the functional activity of macrophages as well as the antimicrobial activities of neutrophils and mononuclear cells^3^. Stimulation of neutrophils, macrophages, and natural killer (NK) cells by β-glucans is evidenced by binding of these cells to dectin-1^4^. Various β-glucans have been extracted from sources such as cereals, yeast, bacteria, and fungi^5^. Among β-glucans, lentinan is extracted from bodies of shiitake (*Lentinus edodes*) and is a high molecular weight polysaccharide^2, 6^. Lentinan, a well known biological response modifier, has a β-(1 → 3) linked backbone and two β-(1 → 6) side chains every five residues^2^. Lentinan enhances the cytotoxic activities of primary macrophages and RAW264.7 cell lines and was found to elevate cytotoxic activity and tumor necrosis factor (TNF) secretion in macrophages both *in vitro* and *in vivo* ^2, 6^. In addition, lentinan has been shown to increase peritoneal macrophage cytotoxicity against metastatic tumors^3^. Moreover, lentinan was shown to have a stimulatory effect on T cells, and lentinan-mediated T cells increase survival of cancer patients^2^. In addition, lentinan has been approved as an adjuvant for gastric cancer patients^7^.

Chronic inflammation is tightly associated with metabolic diseases and carcinogenesis^8, 9^. Inflammasomes, multi-protein complexes, in myeloid cells recognize intracellular pathogen-associated molecular patterns (PAMPs) and/or danger-associated molecular patterns (DAMPs), leading to activation of caspase-1 followed by maturation of pro-inflammatory cytokines and interleukins (IL)-1β and -18^10, 11^. Inflammasomes consist of sensing proteins such as nucleotide-binding oligomerization domain, leucine-rich repeat (NLR) and pyrin domain-containing protein 3 (NLRP3), NLR and caspase activation and recruitment domain (CARD) domain-containing protein 4 (NLRC4), and absent in melanoma 2 (AIM2). In addition, apoptosis-associated speck-like protein containing CARD (ASC) connects sensing proteins and caspase-1^10, 12^. Besides the above canonical inflammasomes, the non-canonical inflammasome activates caspase-11 in mice and caspases-4 and -5 in humans in response to intracellular lipopolysaccharides^13^. The non-canonical inflammasome mediates pyroptosis, endotoxemia, and *Escherichia coli*-induced septic shock as well as triggers NLRP3 inflammasome activation for maturation of IL-1β and -18^13, 14^. Thus, inflammasomes have been progressively studied as a therapeutic target for several diseases^15^.

Lentinan has been reported as a candidate for host-mediated anti-cancer drugs through modulation of the host immune system^16^. Inflammasomes, an intracellular surveillant that triggers the inflammatory response, has been suggested as a therapeutic target for cancer treatment^11^. In this study, we assessed the role of lentinan in canonical (NLRP3, NLRC4, and AIM2) inflammasome activation and inflammasome-mediated gene expression. We further confirmed the effect of lentinan on non-canonical inflammasome activation.

## Results

### Lentinan does not alter NLRP3 inflammasome activation

**Figure 1:**
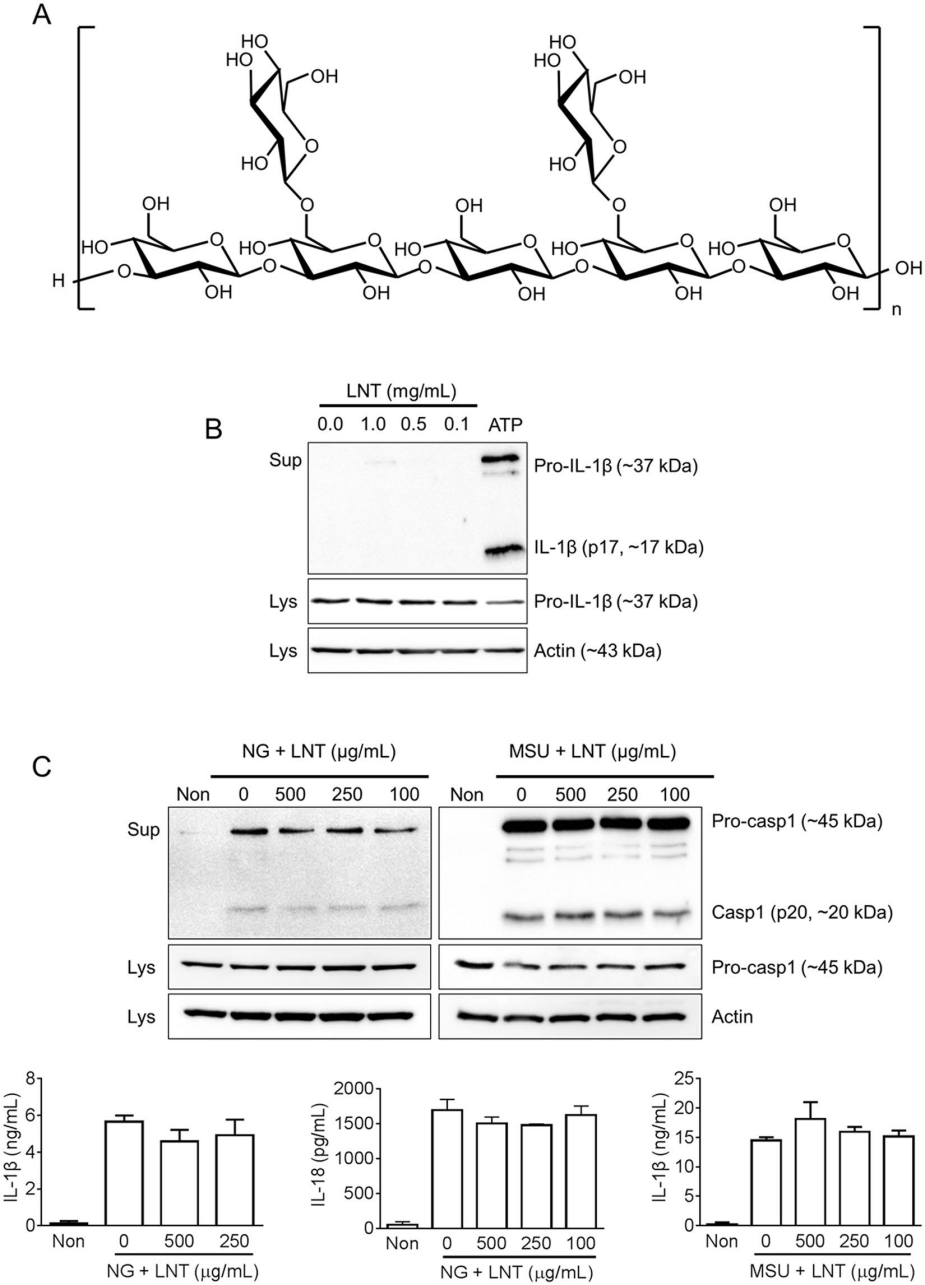
Effect of lentinan on NLRP3 inflammasome activation. (**A**) Chemical structure of lentinan (LNT). (**B**) Lipopolysaccharide-primed bone marrow-derived macrophages (LPS-primed BMDMs) were treated with the indicated concentration of LNT or ATP (2 mM) as a positive control. Secretion of active form of IL-1β was analyzed by immunoblotting. (**C**) LPS-primed BMDMs were treated with the indicated dosage of LNT with/without nigericin (NG, 40 μM) or monosodium urate crystals (MSU, 800 μg/mL). Secretion of caspase-1 (Casp1) was analyzed by immunoblotting, and IL-1β or IL-18 secretion was measured by ELISA. All immunoblot data shown are representative of at least three independent experiments. Bar graph presents the mean ± SD.

Lentinan, a high molecular weight polysaccharide (Fig. 1A), has been reported to induce secretion of inflammatory cytokines such as IL-1β in macrophages^2, 8^. To investigate whether or not lentinan alone can activate inflammasomes resulting in IL-1β maturation and secretion, we treated LPS-primed BMDMs with increasing dosages of lentinan or ATP, a NLRP3 inflammasome trigger, as a positive control. Lentinan alone did not alter IL-1β secretion while ATP did (Fig. 1B). We further determined the effect of lentinan on NLRP3 inflammasome activation. Lentinan did not alter NG- or MSU-mediated IL-1β, IL-18 nor caspase-1 secretion (Fig. 1C). Thus, lentinan could not mediate inflammasome activation nor inhibit NLRP3 inflammasome.

### Lentinan selectively inhibits AIM2 inflammasome activation

**Figure 2:**
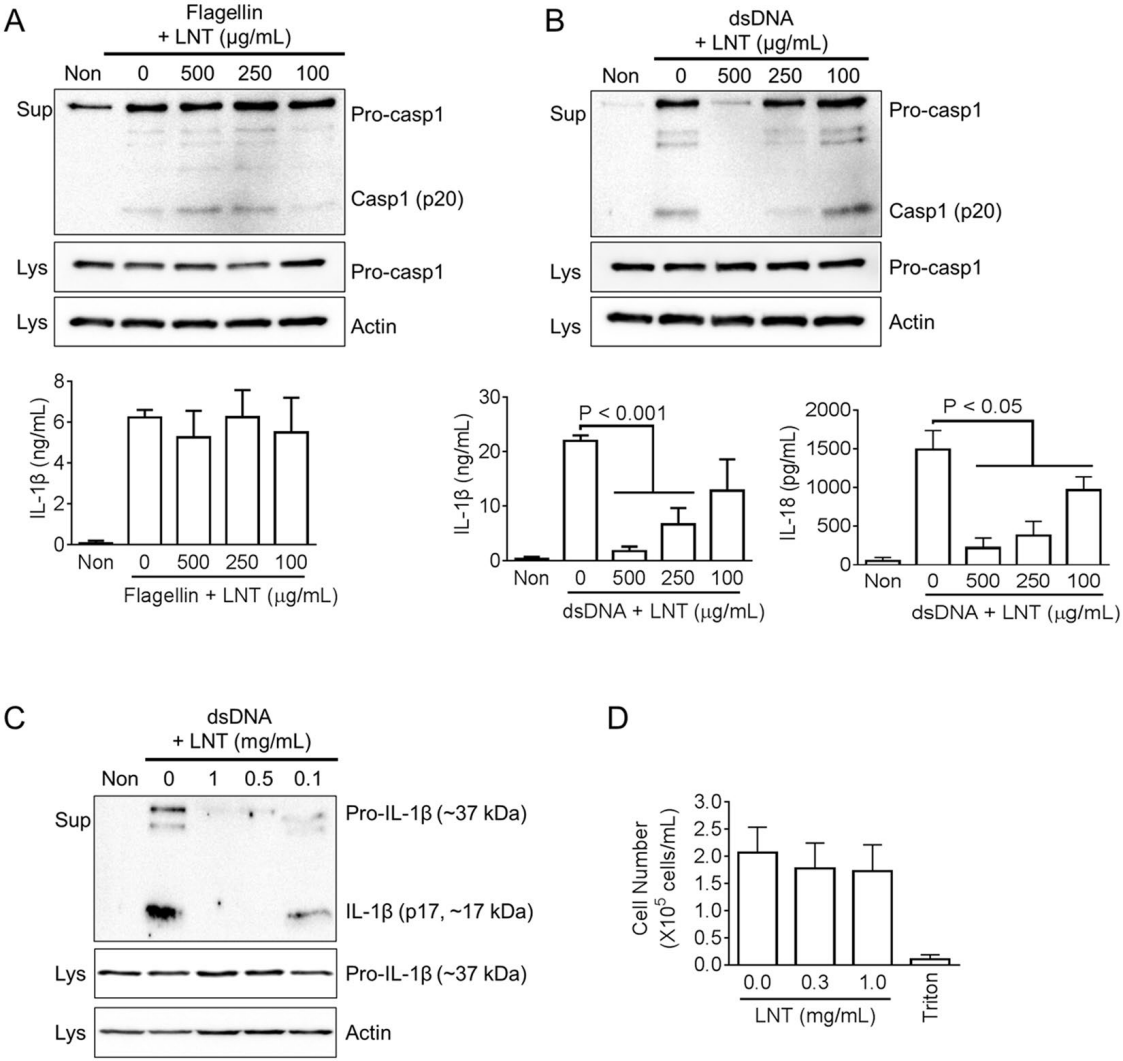
Lentinan on NLRC4 or AIM2 inflammasome activation. LPS-primed BMDMs were treated with the indicated dosage of lentinan (LNT) with/without flagellin (**A**) or dsDNA (**B**). Secretion of caspase-1 (Casp1) was analyzed by immunoblotting, and IL-1β or IL-18 secretion was measured by ELISA. (**C**) Secretion of IL-1β secretion was confirmed by immunoblotting. (**D**) For cytotoxicity, BMDMs were treated with the indicated dosages of LNT, and cell number was measured by an automated cell counter. Triton x-100 (1%, Triton) treatment led to cell death. All immunoblot data shown are representative of at least three independent experiments. Bar graph presents the mean ± SD.

To test the inhibitory effect of lentinan on other inflammasomes such as NLRC4 or AIM2, we transfected flagellin and dsDNA into LPS-primed BMDMs. Similar to NLRP3 inflammasome, lentinan could not regulate secretion of IL-1β or caspase-1 induced by flagellin-mediated NLRC4 inflammasome activation (Fig. 2A). Subsequently, we treated dsDNA-transfected BMDMs with lentinan for AIM2 inflammasome activation. As a result, lentinan dose-dependently inhibited caspase-1, IL-1β, and IL-18 secretion (Fig. 2B). Attenuation of IL-1β secretion was further confirmed by immunoblot assay (Fig. 2C). In addition, we observed that the current lentinan concentration had no cytotoxicity in BMDMs (Fig. 2D). Taken together, lentinan selectively blocks AIM2 inflammasome activation.

### Lentinan up-regulates expression of pro-inflammatory cytokines and inflammasome components via toll-like receptor 4 signaling

**Figure 3:**
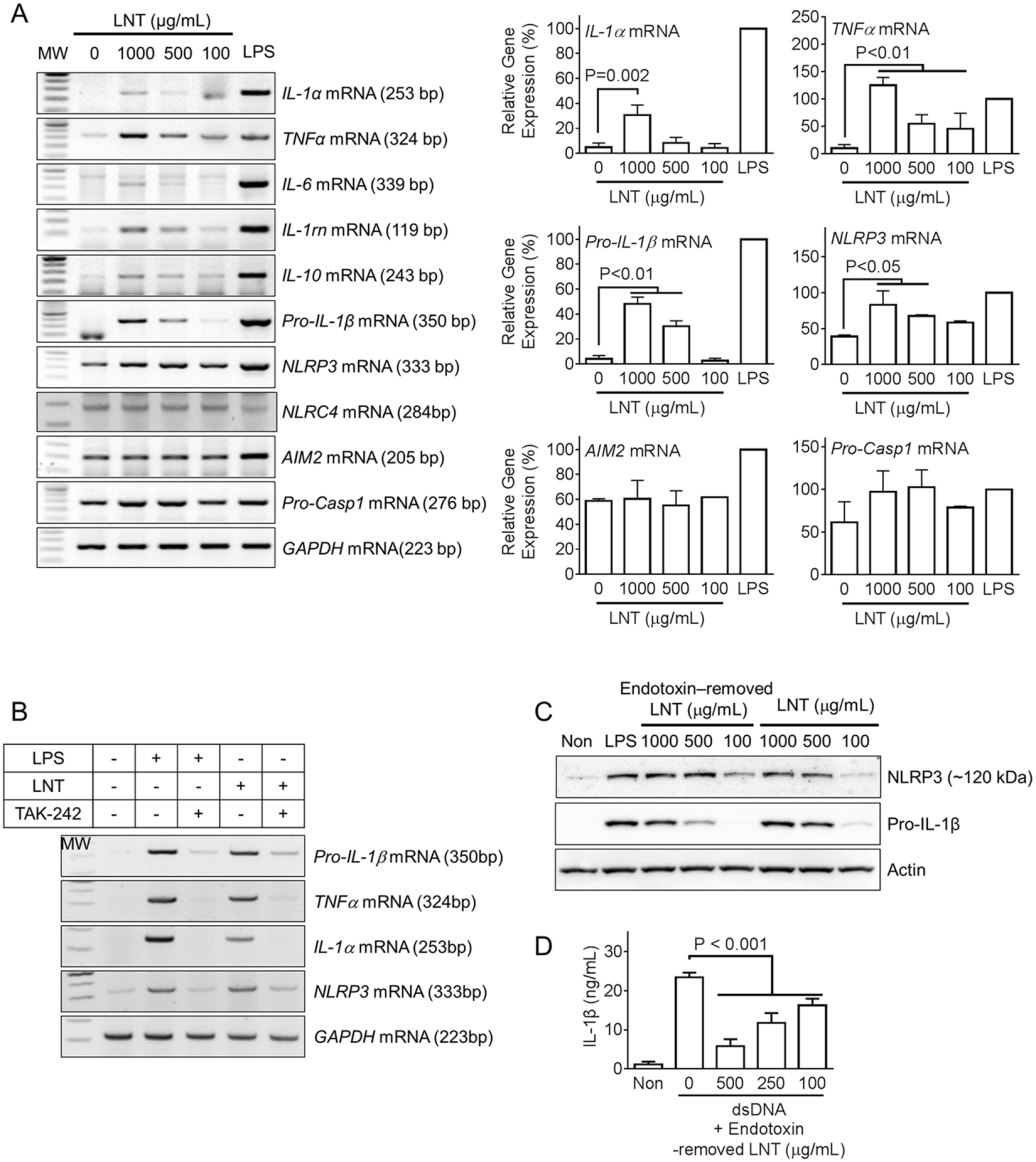
Effect of lentinan on expression of cytokine and inflammasome components. (**A**) BMDMs were treated with the indicated dosage of lentinan (LNT) or LPS (10 ng/mL). Expression of IL-1α, *TNFα*, *IL-6*, *IL-1rn*, *IL-10*, *Pro-IL-1β*, *NLRP3*, *NLRC4*, *AIM2*, and *Pro-Casp1* mRNAs was measured by RT-PCR and the right bar graph indicates relative band density. (**B**) BMDMs were treated with LPS (10 ng/mL) or LNT (1 mg/mL) with/without TAK-242, TLR4 inhibitor. Indicated gene expression levels were assessed by RT-PCR. (**C**) BMDMs were treated with LNT purified by endotoxin removal resin (Endotoxin-removed LNT) or intact LNT as indicated. NLRP3 and Pro-IL-1β proteins were measured by immunoblotting. (**D**) LPS-primed BMDMs were treated with endotoxin-removed LNT with/without dsDNA. Secretion of IL-1β was measured by ELISA. All RT-PCR data shown are representative of at least three independent experiments. Bar graph presents the mean ± SD.

Previously, lentinan has been demonstrated as an inducer of pro-inflammatory cytokines^17^. To determine the effect of lentinan on pro-inflammatory cytokine expression, murine macrophages were treated with increasing dosages of lentinan or LPS, a toll-like receptor 4 (TLR4) ligand, as a positive control. As a result, lentinan treatment alone induced mRNA transcription of inflammatory cytokines such as *IL-1α*, *TNFα*, *IL-6*, *IL-1rn*, *IL-10*, and *Pro-IL-1β* (Fig. 3A). Moreover, inflammasome-related genes were quantitated in response to lentinan treatment. Lentinan increased transcription of *Pro-IL-1β*, *NLRP3*, and *Pro-Casp1* but had no effect on *NLRC4* and *AIM2* (Fig. 3A). Next, we determined which pathway mediates lentinan-induced gene expression. Based on a previous report^18^ that lentinan up-regulates TLR2 and 4 expression, treatment with TAK-242, a TLR4 signaling inhibitor, resulted in reduction of cytokine production and *NLRP3* mRNA expression in response to lentinan (Fig. 3B). In addition, we purified LNT with endotoxin removal resin and measured endotoxin contamination by LAL assay. Endotoxin contamination levels were not significantly different between purified LNT (0.4077 ± 0.0006 EU/mg) and intact LNT (0.4345 ± 0.0008 EU/mg). We further compared expression levels of NLRP3 and Pro-IL-1β proteins between purified and intact LNT (Fig. 3C). As a result, purified LNT induced NLRP3 and Pro-IL-1β expression similar to intact LNT. Moreover, purified LNT presented similar anti-AIM2 inflammasome properties (Fig. 3D). Taken together, lentinan stimulates production of pro-IL-1β and NLRP3 through TLR4 signaling but inhibits maturation of IL-1β induced by AIM2 inflammasome activation.

### Lentinan induces priming step of NLRP3 inflammasome activation and blocks formation of Asc pyroptosome

**Figure 4:**
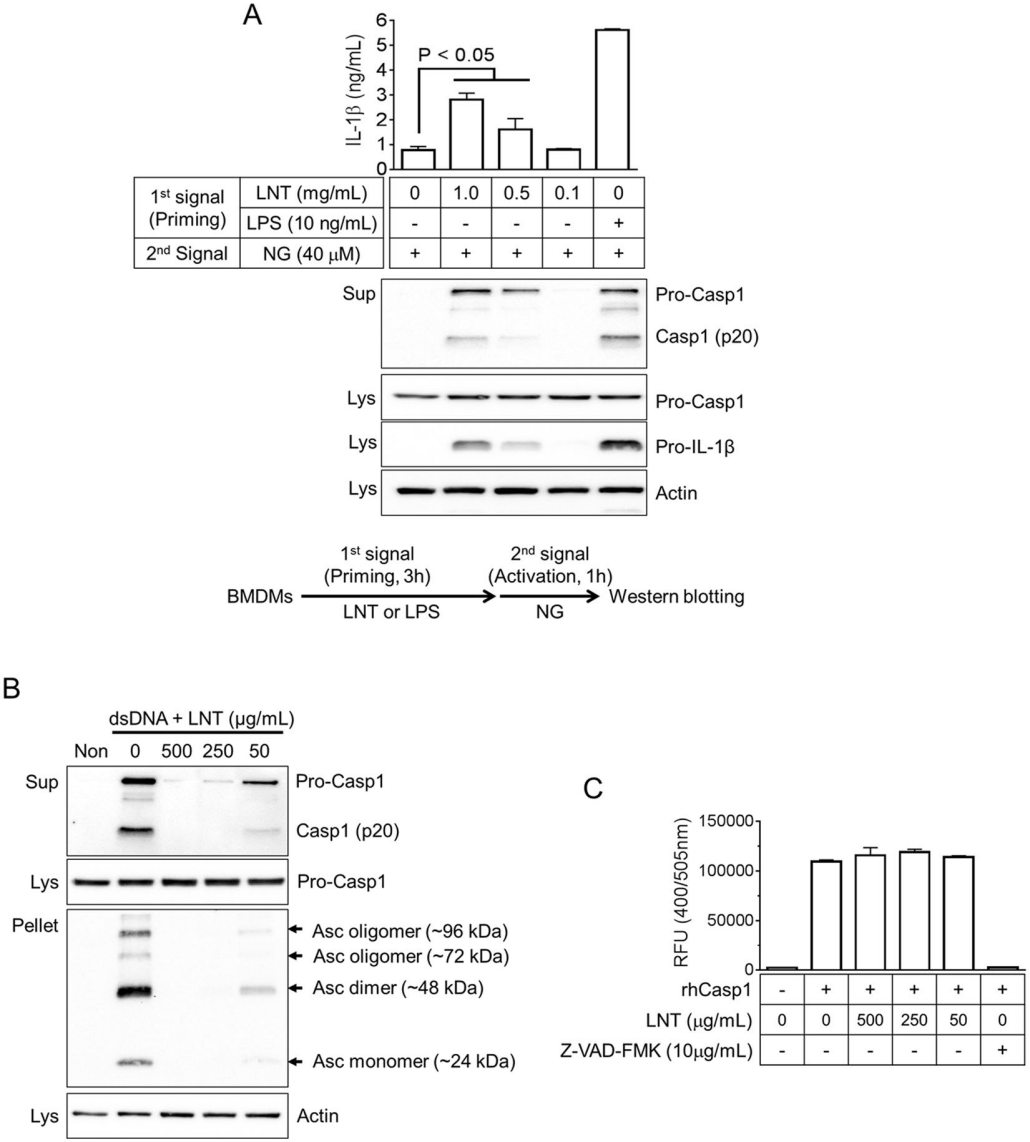
Effect of lentinan on priming step of inflammasome activation and formation of Asc pyroptosome. (**A**) BMDMs were treated with lentinan (LNT) or LPS as the 1^st^ signal, after which cells were replaced by media containing nigericin (NG, 2^nd^ signal). Secretion of IL-1β was measured by ELISA, and Casp1 secretion and pro-IL1β expression were analyzed by immunoblotting. (**B**) LPS-primed BMDMs were transfected with dsDNA in the presence of LNT. Casp1 secretion and Asc pyroptosome formation were analyzed by immunoblotting using cell culture supernatants (Sup), cell lysates (Lys), and cross-linked pellets (Pellet) from whole cell lysates. (**C**) Recombinant human caspase-1 (rhCasp1) was incubated with its substrate (YVAD-pNA) in the presence of LNT as indicated. Bar graph presents the mean ± SD of relative fluorescence unit (RFU).

Activation of NLRP3 inflammasome requires two steps, priming and activation. The priming step is commonly mediated by toll-like receptor (TLR) ligands such as LPS, which triggers NF-κB signaling to induce transcription of pro-IL-1β and NLRP3^19^. Based on Fig. 3, LNT could act as a priming agent for NLRP3 inflammasome due to LNT-mediated transcription and translation of pro-IL-1β and NLRP3 via TLR4 signaling. Thus, we tested the effect of LNT on priming of inflammasome activation. As shown in Fig. 4, LNT-primed BMDMs showed IL-1β and Casp1 secretion in response to NG, a NLRP3 inflammasome trigger, similar to LPS-primed BMDMs. Taken together, LNT had an adequate effect on the priming step of NLRP3 inflammasome activation.

Next, we assessed the effect of LNT on formation of Asc pyroptosome and *in vitro* activation of caspase-1 to determine the putative mechanism for impairment of AIM2 inflammasome by LNT. Based on the literature^20^, AIM2 recognizes intracellular dsDNA and then interacts with ASC, which leads to the formation of Asc pyroptosome. The pyroptosome then activates caspase-1 and induces IL-1β secretion^20^. As shown in Fig. 4B, LNT dose-dependently attenuated formation of Asc pyroptosome, similar to secretion of Casp-1. In addition, we observed the effect of LNT on recombinant human caspase-1 (rhCasp1) activities (Fig. 4C). Activity of rhCasp-1 was blocked by the pan-capase-1 inhibitor Z-VAD-FMK but not by LNT. Thus, these data indicate that LNT disrupts pyroptosome formation upstream.

### β-D-Glucan extracted from barley does not regulate cytokine production and maturation

We next investigated whether or not β-glucan originating from different sources can regulate inflammasome activation and gene expression. β-D-Glucan extracted from barley (GB) has a structure consisting of β-(1,3)-glucose units (Supplementary Fig. 1A). Similar to lentinan, GB alone did not induce IL-1β secretion in LPS-primed BMDMs (Supplementary Fig. 1B). Next, we treated LPS-primed macrophages with GB in the presence of NG for activation of NLRP3 inflammasome, flagellin for activation of NLRC4 inflammasome, or dsDNA for activation of AIM2 inflammasome. As a result, inflammasome activations were not inhibited by GB co-treatment (Supplementary Fig. 1C). In addition, murine macrophages did not induce pro-inflammatory cytokine production in response to GB treatment (Supplementary Fig. 1D). Thus, the inflammasome-regulatory properties of β-glucans vary depending on the source.

### Lentinan reduces Listeria-mediated IL-1β secretion in mice

**Figure 5:**
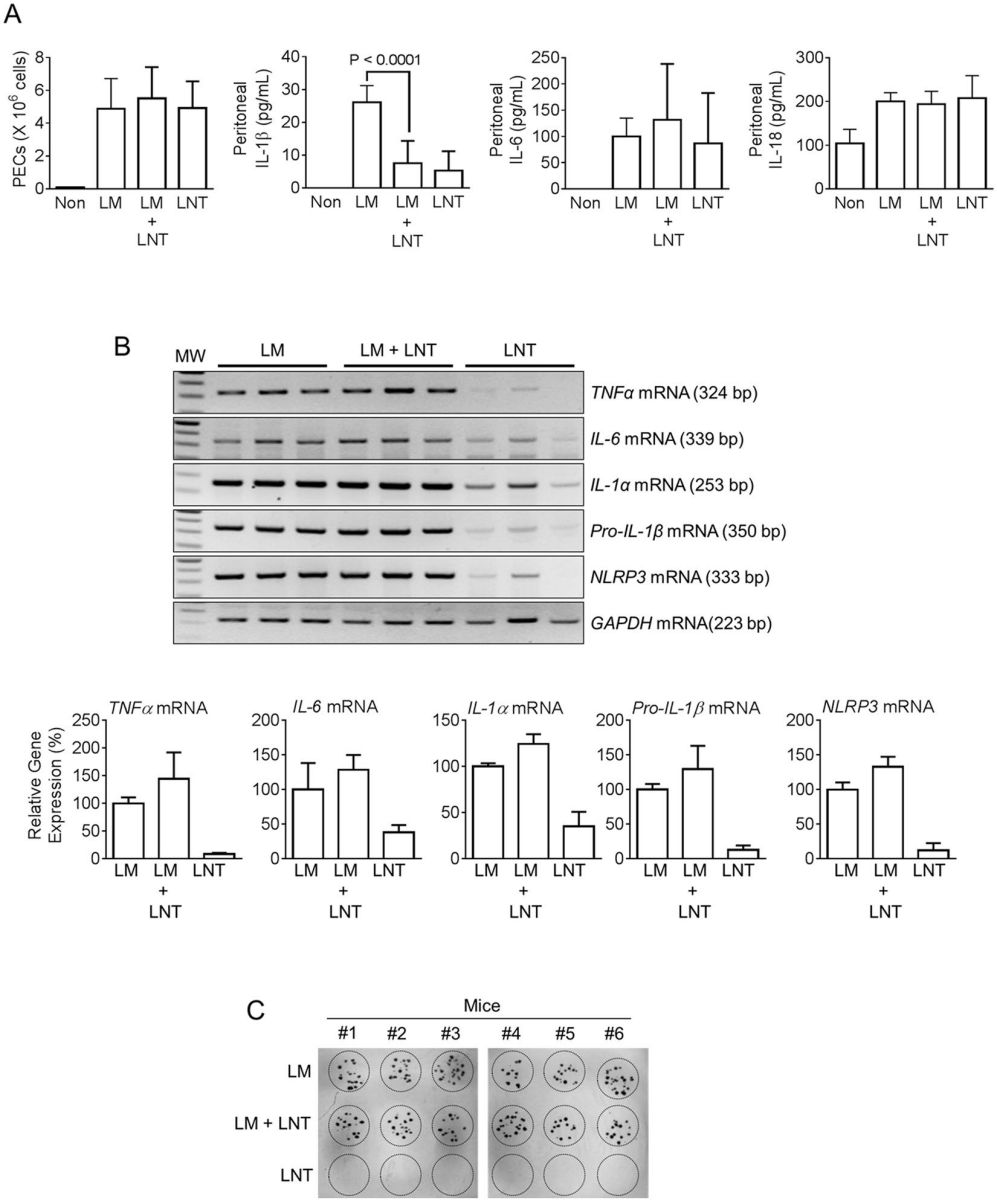
Effect of lentinan on *Listeria monocytogenes* (LM)-induced peritonitis. (**A**) Mice (n = 3 for non-treated, n = 14 for LM- or LM+LNT−, and n = 6 for LNT-treated group) were ip-injected with *Listeria monocytogenes* (1,000 cfu in 200 μL of saline) with/without lentinan (LNT, 10 mg/kg). Number of peritoneal exudate cells (PECs) was calculated, and IL-1β, IL-6, and IL-18 secretion levels of peritoneal lavage fluids measured. (**B**) PECs were isolated from LM- and/or LNT-treated mice (n = 3 for group), and expression levels of *TNFα*, *IL-6*, IL-1α, *Pro-IL-1β*, and *NLRP3* mRNAs were measured by RT-PCR. The bar graph (mean ± SD) indicates relative band density. (**C**) Peritoneal lavage fluids were collected from LM- and/or LNT-treated mice (n = 6 per group), and 10 μL of lavage was cultured onto BHI plates to assess peritoneal LM burden.

In the current study, lentinan exhibited conflicting effects on inflammatory responses. Lentinan up-regulated pro-inflammatory cytokines but blocked IL-1β secretion mediated by AIM2 inflammasome activation. To assess the role of lentinan on AIM2 inflammasome activation, we adopted *Listeria monocytogenes* (LM)-induced peritonitis. LM has been revealed as an AIM2 inflammasome trigger, although it also activates NLRP3 and NLRC4 inflammasomes^21–23^. Mice injected with LM presented increased numbers of peritoneal exudate cells (PECs) as well as elevated peritoneal IL-1β, IL-6, and IL-18 secretion (Fig. 5A). Lentinan treatment significantly attenuated LM-induced IL-1β secretion but not PECs, IL-6, or IL-18 secretion. These data suggest that lentinan has anti-AIM2 inflammasome properties. However, we further investigated the priming effect of lentinan *in vivo*. For this, we isolated peritoneal exudate cells (PECs) from LM- and/or LNT-injected mice and analyzed the transcription levels of pro-inflammatory cytokines and inflammasome components. As shown in Fig. 5B, LM and/or LNT treatments up-regulated expression of *TNFα*, *IL-6*, *IL-1α*, *pro-IL-1β*, and *NLRP3* mRNAs in PECs, although the expression levels were lower in LNT alone treatment. This result implies that LNT selectively blocked IL-1β maturation resulting from inflammasome activation, although LNT up-regulated transcription of *pro-IL-1β* mRNA in PCEs. The increased PECs and IL-6 secretion (Fig. 5A) might have been mediated by LNT-mediated cytokine up-regulation. However, we cannot verify the different regulatory pathways of IL-18 secretion upon LNT treatment *in vivo* and *in vitro* at this moment. In addition, we tested the effect of LNT on peritoneal LM burden. As shown in Fig. 5C, LNT treatment did not alter the number of peritoneal LM, implying that LNT inhibited peritoneal IL-1β secretion in LM-infected mice without alteration of bacterial burden.

### Lentinan alleviates lethality of endotoxemia in mice

**Figure 6:**
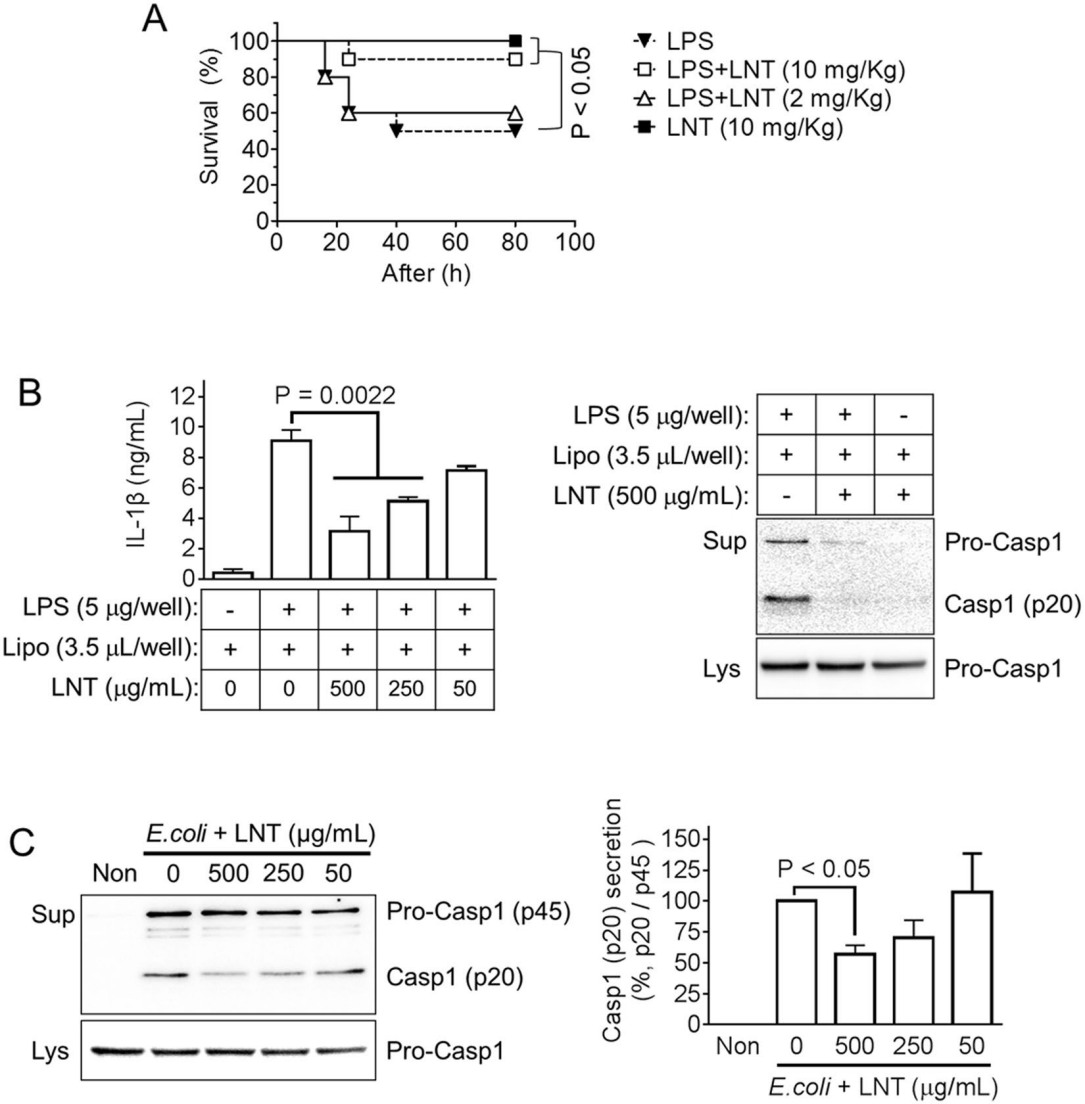
Effect of lentinan on endotoxemic models. (**A**) Mice (n = 10 per group) were intraperitoneally (ip) injected with LPS (25 mg/kg) and/or lentinan (LNT) as indicated. Survival rates were observed at the indicated times. (**B**), LPS-primed BMDMs were transfected with LPS to activate non-canonical inflammasome in the presence of LNT as indicated. IL-1β and Casp1 secretion was analyzed by ELISA and immunoblotting. (**B**) LPS-primed BMDMs were infected with *E*. *coli*. (MOI = 10) to activate non-canonical inflammasome in the presence of LNT as indicated. Secretion of Casp1 was analyzed by immunoblotting, and the right bar graph indicates relative band density. Bar graphs present the mean ± SD. Lipo, Lipofectamine 2000^TM^.

Next, we focused on LPS-induced septic shock, also called endotoxemia. The LPS-induced septic shock model is a well characterized model of NLRP3 inflammasome-mediated disease, and intracellular LPS is known to trigger non-canonical inflammasome activation^24, 25^. As seen in Fig. 6A, mice injected with LPS alone presented 50% lethality while mice administrated lentinan alone did not show any mortality. Notably, lentinan injection into LPS-treated mice significantly reduced lethality in a dose-dependent manner. We further tested the effect of LNT on non-canonical inflammasome activation since LPS lethality is tightly involved in the non-canonical inflammasome^14^. We performed LPS transfection and *E*. *coli* infection into BMDMs to trigger non-canonical inflammasome activation, which activates caspase-11 and then triggers NLRP3 inflammasome activation for Casp1 and IL-1β secretion^13, 14^. As shown in Fig. 6B, LPS transfection induced IL-1β secretion which was blocked by LNT treatment. In addition, LNT attenuated Casp1 secretion resulting from *E*. *coli*-mediating non-canonical inflammasome activation (Fig. 6C). This suggests that LNT ameliorated endotoxemic lethality due to LNT-mediated inhibition of non-canonical inflammasome activation.

## Discussion

In this study, we confirmed the effect of lentinan, β-glucan from shiitake, on inflammasome activation and cytokine maturation. As a result, lentinan selectively inhibited IL-1β maturation in response to AIM2 inflammasome activation in murine macrophages, whereas NLRP3 and NLRC4 inflammasomes were not activated by lentinan treatment. As expected, lentinan up-regulated pro-inflammatory cytokines and stimulated TLR4 signaling, resulting in expression of the inflammasome components NLRP3 and pro-IL-1β. Unlike lentinan, β-D-glucan extracted from barley (GB) did not alter cytokine maturation nor expression in macrophages. Furthermore, lentinan ameliorated LPS-induced lethality in a dose-dependent manner and selectively attenuated IL-1β secretion in response to *Listeria monocytogenes*-mediated AIM2 inflammasome activation. Taken together, lentinan stimulates expression of pro-inflammatory cytokines, including NLRP3, whereas it selectively attenuates cytokine maturation in response to AIM2 inflammasome activation.

The effects of β-glucans on inflammation activation have been previously studied^26, 27^. Curdlan, a β-glucan from *Alcaligenes faecalis*, was shown to up-regulate *Pro-IL-1β* mRNA expression via interaction with dectin-1 as well as induce NLRP3 inflammasome-mediated IL-1β secretion resulting from NLRP3 inflammasome activation through dectin-1/Syk signaling^26^. However, other β-glucans such as GB, paramylon, and zymosan did not modulate IL-1β secretion^26^. In the current study, lentinan regulated cytokine production and maturation while GB did not, implying β-glucans had various effects on inflammasome activation depending on source or chemical structure. The effect of lentinan on NLRP3 inflammasome activation has been previously studied, whereas the previous study used human lung cancer cell lines, A549, instead of macrophages or dendritic cells^27^. Co-treatment of lentinan with paclitaxel, an anti-cancer drug, to A549 cells induces reactive oxygen species (ROS) production and thioredoxin-interacting protein (TXNIP) expression, which are tightly associated with NLRP3 inflammasome activation^28^. Further, co-treatment of lentinan with paclitaxel has been shown to stimulate the TXNIP-NLRP3 interaction, leading to IL-1β and Casp1 maturation^28^. However, we did not observe IL-1β secretion from LPS-primed BMDMs in response to lentinan alone (Fig. 1B). Thus, the inflammasome-modulating properties of lentinan may vary depending on cell type or chemical combination.

Lentinan as a strong active macromolecule is known to enhance host-mediated anti-cancer activities in the human immune system^29^. For example, cancer patients administered lentinan present a higher survival rate due to enhanced antibody- and complement-dependent cytotoxicity against tumor cells^16^. In addition, lentinan administration has been shown to induce generation of cytotoxic T cells and NK cells, stimulating their anti-cancer activities^16^. Furthermore, lentinan was shown to stimulate cytotoxicity of macrophages against metastatic tumors in mouse studies^30^. Thus, lentinan-stimulated immunity is a key mechanism responsible for its anti-cancer properties. In this study, we observed that lentinan induced cytokine and NLRP3 expression but inhibited AIM2 inflammasome activation in macrophages. Although NLRP3 and NLRC4 inflammasomes are involved in carcinogenesis and anticancer immune responses^11^, AIM2 inflammasome has not been reported to induce cancer development. Instead of inflammasome activation, loss of *AIM22* gene has been shown to result in cancer growth while mutation of *AIM2* is associated with development of various cancers in humans^31, 32^. Moreover, Aim2^−/−^ mice showed increased size and number of colon tumors in a colitis-associated cancer (CAC) model, implying that the AIM2 gene itself regulates tumor progress and prevents colorectal cancer^33^. Based on the literature, we conclude that the anti-cancer properties of lentinan are not mediated by inflammasomes since lentinan did not alter AIM2 mRNA expression nor activation of NLRP3 and NLRC4 inflammasomes.

AIM2 contains a pyrin domain, which interacts with ASC, as well as hematopoietic interferon-inducible nuclear protein (HIN)-200 domains, which sense cytosolic dsDNA^15^. These domains form an auto-inhibitory conformation before detection of the trigger while auto-inhibition is relieved by cytosolic dsDNA, resulting in caspase-1-dependent pyroptosis and release of IL-1β and -18. AIM2 inflammasome activation is critical for host defense against DNA viruses and bacteria that replicate in the cytosol^34^. For example, *Listeria monocytogenes* predominantly activates AIM2 inflammasome among several inflammasomes^22, 35^. In addition, *Listeria monocytogenes*-triggered pyroptosis and cytokine secretion were shown to be diminished in Aim2^−/−^ macrophages^22^. However, *Listeria monocytogenes* trigger AIM2 inflammasome activation as well as activation of NLRP3 and NLCR4 inflammasomes. That is, NLRP3 inflammasome is activated by lysosomal disruption when *Listeria monocytogenes* escape to the cytoplasm of phagocytes^22^. In addition, *Listeria* are critical for NLRC4 inflammasome activation in the absence of LPS priming^23^, whereas LPS-primed macrophages derived from NLRC4^−/−^ mice do not respond to *Listeria* infection for inflammasome activation due to LPS priming bypassing the requirement for NLRC4^22^. Although multi-inflammasomes are involved in *Listeria* infection, AIM2 is the most accepted inflammasome against *Listeria monocytogenes* ^22, 23, 35^. *Francisella tularensis*, which causes tularemia, triggers activation of AIM2 inflammasome, leading to IL-1β and 18 production in infected macrophages^36^. AIM2 inflammasome is also crucial for intestinal homeostasis^37^. In Aim2^−/−^ mice, intestinal epithelial cells (IEC) show diminished IL-18 secretion, which is required for IEC proliferation and tissue regeneration, during steady state^37, 38^. In contrast, over-production of cytokines induced by chronic inflammasome activation leads to tissue damage and colitis-associated colorectal cancer^39^. Previously, oral administration of lentinan was shown to ameliorate susceptibility to dextran sulfate sodium (DSS)-induced colitis and attenuate intestinal inflammation^3^. Although we did not confirm the effect of lentinan in DSS-induced colitis, a well characterized inflammasome-mediated disease, lentinan might block IL-1β and -18 secretion in a colitis model^38^. Thus, lentinan is suggested as an anti-inflammasome agent, especially for AIM2 inflammasome.

## Materials and Methods

### Preparation of bone marrow-derived macrophages (BMDMs)

BMDMs were obtained by differentiating bone marrow progenitors from the tibia and femur bones of C57BL/6 mice (6- to 12-weeks-old; Narabio Co., Seoul, Korea) in the presence of macrophage colony-stimulating factor (M-CSF) secreted from L929 cells^40–42^. Bone marrow cells were cultured in RPMI 1640 supplemented with 10% fetal bovine serum (FBS, GenDEPOT Inc., Barker, TX, USA), 50% M-CSF containing media from L929 cells, 100 U/mL of penicillin, and 100 µg/mL of streptomycin. Cells were plated in non-tissue culture-treated Petri dishes (SPL Life Science Co., Phocheon-si, Gyeonggi-do, Korea) and incubated at 37 °C in 5% CO_2_ atmosphere for 7 days.

### Cell treatment

For inflammasome activation, BMDMs (1.0 × 10^6^ cells per well) in RPMI 1640 containing 10% FBS and antibiotics were seeded in 12-well tissue culture plates (SPL Life Science Co.) and primed with lipopolysaccharide (LPS, 1 μg/mL; #L4130, Sigma-Aldrich Co., St. Louis, MO, USA)^43, 44^. After 3 h, LPS-primed BMDMs were given fresh media (RPMI 1640, 350 μL in 12-well plates) without FBS or antibiotics in the presence of adenosine triphosphate (2 mM, ATP; #tlrl-atp, InvivoGen, San Diego, CA, USA) for 1 h, nigericin (40 μM, NG; #4312 Tocris Bioscience, Bristol, UK) for 1 h, flagellin (0.5 μg/mL; #tlrl-stfla, InvivoGen) with Lipofectamine 2000 (10 μL/mL, Invitrogen, Grand Island, NY, USA) for 1 h, double-stranded DNA (1 μg/mL, dsDNA) with jetPRIME^TM^ (2 μL/mL, Polyplus-transfection Inc., Illkirch, France) for 1 h, monosodium urate crystals (800 μg/mL, MSU, Sigma-Aldrich Co.) for 6 h, LPS (14 μg/mL) with Lipofectamine 2000 (10 μL/mL) for 6 h, or *Escherichia coli* (*E*. *coli*, DH5α, multiplicity of infection [MOI] = 10, Invitrogen) for 6 h.

Lentinan (LNT, #FL33321, Carbosynth, Compton, Berkshire, UK) was treated with inflammasome triggers at the same time. LNT was purified by Pierce^TM^ High-Capacity Endotoxin Removal Resin (#88270, ThermoFisher Scientific, Waltham, MA, USA) according to the manufacturer’s protocol. In addition, endotoxin contamination of the purified and intact LNT was tested with LAL assay kit (#QCL-1000, Lonza Group Ltd., Basel, Switzerland).

For gene expression, BMDMs (2.0 × 10^6^ cells per well) were plated in 6-well plates (SPL Life Science Co.) and treated with LNT or LPS (10 ng/mL, Sigma-Aldrich Co.) for 3 h. In addition, cells were treated with LNT or LPS in the presence of TAK-242 (5 μM, #CLI-095, InvivoGen) for 3 h.

### Western blot analysis

For analysis of inflammasome activation, cellular supernatant (Sup) was collected, and remaining cells were lysed with lysis buffer containing proteinase inhibitor cocktail (#M250-1, AMRESCO LLC, Solon, OH, USA). The lysate (Lys) was transferred into a new tube and collected by centrifugation at 15,000 rcf for 5 min. The remaining pellet was washed two times with PBS and then re-suspended and cross-linked with 2 mM suberic acid bis (Sigma-Aldrich Co.) for 1 h, followed by centrifugation at 15,000 rcf for 5 min. The cross-linked pellets (Pellet) were re-suspended in 50 μL of 2 X loading dye buffer (116 mM Tris, 3.4% SDS, 12% glycerol, 200 mM DTT, 0.003% bromo phenol blue)^12, 36^. The Sup, Lys, and Pellet were subjected to Western blot assay. Unless otherwise indicated, all materials for Western blot analysis were purchased from BIO-RAD (Hercules, CA, USA). Sup, Lys, or pellet were separated by SDS-PAGE (10% or 16%) using running buffer and transferred onto a polyvinylidene difluoride membrane (PVDF; #10849 A, Pall Co., Port Washington, NY, USA) using transfer buffer. The membranes were blocked with 3% skim milk and probed overnight at 4 °C with anti-mouse IL-1β antibody (#AF-401-NA, R&D Systems, Minneapolis, MN, USA), anti-caspase-1 antibody (#AG-20B-0042, AdipoGen Co., San Diego, CA, USA), anti-NLRP3 antibody (#AG-20B-0014-C100, AddipoGen Co.), anti-Asc antibody (#sc-22514-R, Santa Cruz Biotechnology, Santa Cruz, CA, USA) or anti-actin antibody (#sc-1615, Santa Cruz Biotechnology). Membranes were further probed with HRP-conjugated 2^nd^ anti-sera (#sc-2020 or #sc-2004, Santa Cruz Biotechnology) and visualized by Power-Opti ECL^TM^ solution (BioNote Co., Hwasung-si, Gyeonggi-do, Korea) and a cooled CCD camera System (#AE-9105, EZ-Capture^TM^ II, ATTO Technology, Tokyo, Japan). The details of immunoblotting buffers were described in previous studies^44, 45^.

### Animal experiments

Male C57BL/6 mice (8- to 10-weeks-old) were purchased from Narabio Co. (Seoul, Korea). All mice were maintained under a 12 h light/dark cycle at 24 °C. Animals were provided standard sterile food and water *ad libitum*, after which they were allowed to adjust to the environment for 1 week. Mice were intraperitoneally (ip) injected with lentinan (2 or 10 mg/kg, Carbosynth) after 1 h of LPS (25 mg/kg, Sigma-Aldrich Co.) or saline (200 μL) ip treatment. Mouse mortality was observed every 8 h for 4 days. Mice were ip-injected with lentinan (10 mg/kg) after 1 h of *Listeria monocytogenes* (1,000 cfu per mouse) ip treatment. After 5 h, mice were sacrificed by CO_2_ inhalation. Peritoneal cavities were washed with 5 mL of PBS, and peritoneal exudate cells (PECs) were analyzed by a cell counter (Moxi^TM^ Z Mini Automated Cell Counter, ORFLO Technologies, Ketchum, ID, USA). Lavage fluids were collected for further analysis. All animal experiments were carried out in accordance with the National Institutes of Health Guide for the Care and Use of Laboratory Animals and approved by the Institutional Animal Care and Use Committee of Kangwon National University (IACUC; approval no. KW-160114-1).

### Reverse transcription polymerase chain reaction (RT-PCR)

Total RNA was extracted using NucleoZOL (MACHEREY-NAGEL GmbH & Co. KG, Postfach, Düren, Germany) and reverse-transcribed to first-strand complementary DNA (cDNA) using an M-MLV cDNA Synthesis kit (Enzynomics, Daejeon, Korea). Transcription was amplified using a SimpliAmp^TM^ Thermal Cycler (Thermo Fisher Scientific Inc. Grand Island, NY, USA) and nTaq polymerase (Enzynomics). PCR products were visualized by agarose gel electrophoresis, ethidium bromide staining, and EZ-Capture^TM^ II (ATTO Technology). Band intensity was measured by CS Analyzer Version 3.00 (ATTO Technology). Gene-specific primers are as follows. Pro-IL-1β (*Il1b*; Genebank ID: NM_008361) primers 5′-CCC AAG CAA TAC CCA AAG AA-3′ and 5′-GCT TGT GCT CTG CTT GTG AG-3′; TNFα (*Tnfa*; NM_013693) 5′-ACG GCA TGG ATC TCA AAG AC-3′ and 5′-GTG GGT GAG GAG CAC GTA GT-3′; IL-1α (*Il1a*; NM_010554) 5′-CCG ACC TCA TTT TCT TCT GG-3′ and 5′-GTG CAC CCG ACT TTG TTC TT-3′; NLRP3 (*Nlrp3*; NM_145827) 5′-CAG GCG AGA CCT CTG GGA AA-3′ and 5′-CCC AGC AAA CCC ATC CAC TC-3′; NLRC4 (*Nlrc4*; NM_001033367) 5′-ATC GTC ATC ACC GTG TGG AG-3′ and 5′-CTT CAG CAG CAG AGC CTT GA-3′; AIM2 (*Aim2*; NM_001013779) 5′-AGT GGC CAC GGA GAC AGA TT-3′ and 5′-GGG AGT TTC CCT GGC TCT CT-3′; Pro-Caspase-1 (*Casp1*; NM_009807) 5′-CTG GGA CCC TCA AGT TTT GC-3′ and 5′-GGC AGG CAG CAA ATT CTT TC-3′; GAPDH (*Gapdh*; NM_001289726) 5′-AAC TTT GGC ATT GTG GAA GG-3′ and 5′-ACA CAT TGG GGG TAG GAA CA-3′.

### IL-1β and IL-18 detection using ELISA

To quantitate secreted IL-1β or IL-18, cell culture supernatants of BMDMs or peritoneal lavage fluids were measured by a mouse IL-1beta/IL-1F2 Quantikine ELISA Kit (R&D Systems) or mouse IL-18 platinum ELISA (eBioscience, San Diego, CA, USA). ELISA plates were readout using an Epoch^TM^ microplate spectrophotometer (BioTek, Winooski, VT, USA).

### Bacterial growth

*Listeria monocytogenes* from the Korean Culture Center of Microorganisms (KCCM; Seoul, Korea) and *E*. *coli* (DH5α, Invitrogen) were grown on Brain Heart Infusion (BHI, Laboratories Conda, Madrid, Spain) and Luria-Bertani (LB, Laboratories Conda) broth for 18 h with shaking at 37 °C or plated on a BHI or LB plate to calculate colony-forming units (cfu). For assessment of bacterial burden, peritoneal lavage (10 μL) was dropped onto BHI plates and incubated overnight.

### Cytotoxicity assay

BMDMs (5 × 10^5^ cells per well) were plated in a 6-well plate (SPL Life Science Co.) and treated with lentinan 0, 0.3, or 1.0 mg/mL. After 3 h, cells were lifted by a scraper in cold PBS and analyzed by a cell counter (Moxi^TM^ Z Mini Automated Cell Counter).

### Caspase-1 activity assay

Recombinant human caspase-1 (1 unit/rx, #1081, BioVision Inc., CA. USA) was incubated with YVAD-pNA, a substrate of caspase-1, in the presence of lentinan or Z-VAD-FMK (FMK001, R&D Systems). Caspase-1 activity was measured using a Caspase-1/ICE Fluorometric Assay Kit (#K110, BioVision Inc.) according to the manufacturer’s protocol.

### Statistical analyses

Statistical analyses were performed using a t-test (Mann-Whitney test) for the two groups, one-way ANOVA (Turkey’s multiple comparisons test) for multiple groups, and log-rank (Mantel-Cox) test for lethality assessment using GraphPad Prism 6 (GraphPad Software, San Diego, CA, USA).

## Electronic supplementary material

Supplementary Information
