# Supplementary material for: Lentinan from shiitake selectively attenuates AIM2 and non-canonical inflammasome activation while inducing pro-inflammatory cytokine production
Source: Sci Rep. 2017 May 2;7:1314. doi: 10.1038/s41598-017-01462-4 (PMC5431005; doi:10.1038/s41598-017-01462-4)
Supplement: Supplementary file 1 — Supplementary Information [file 41598_2017_1462_MOESM1_ESM.pdf]

Supplementary Information

Lentinan from shiitake selectively attenuates AIM2 and non-canonical inflammasome activation while inducing pro-inflammatory cytokine production

Huijeong Ahn[a#], Eunsaem Jeon[a#], Jin-Chul Kim[b], Seung Goo Kang[c], Sung-il Yoon[d], Hyun-Jeong Ko[e], Pyeung-Hyeun Kim[c], and Geun-Shik Lee [a]*

[a]Laboratory of Physiology and Inflammatory Diseases, College of Veterinary Medicine and Institute of Veterinary Science, Kangwon National University, Chuncheon 24341, Republic of Korea

[b]Natural Products Research Center, Korea Institute of Science and Technology, Gangneung 25451, Republic of Korea.

[c]Department of Molecular Bioscience, School of Biomedical Science, Kangwon National University, Chuncheon 24341, Republic of Korea.

[d]Division of Biomedical Convergence, College of Biomedical Science, Kangwon National University, Chuncheon 24341, Republic of Korea.

[e]Laboratory of Microbiology and Immunology, College of Pharmacy, Kangwon National University, Chuncheon 24341, Republic of Korea.

Supplemental figure 1.

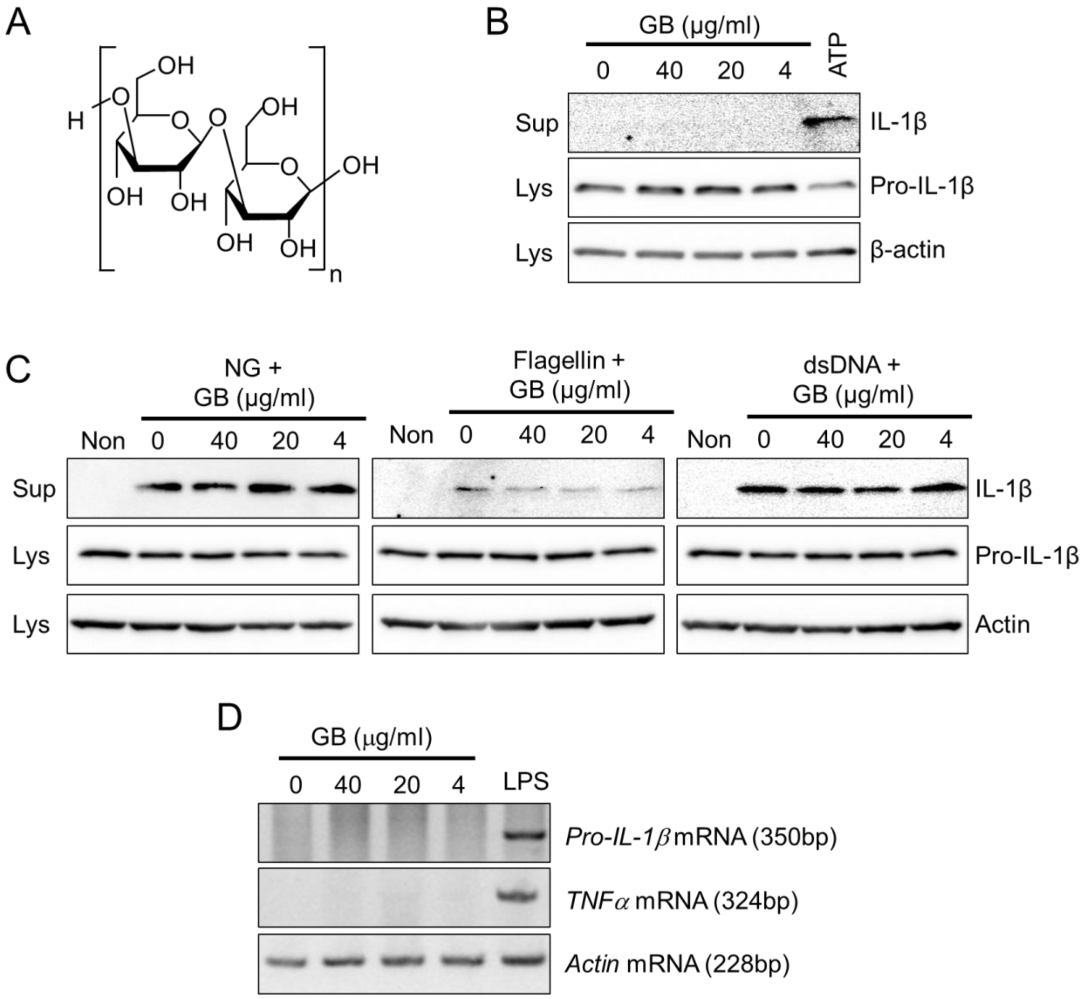

Supplemental figure 1. β-D-Glucan extracted from barley on cytokine production and maturation.

A, Chemical structure of β-D-glucan extracted from barley (GB, #G6513, Sigma-Aldrich Co.). **B**, LPS-primed BMDMs were treated with the indicated concentration of GB or ATP (2mM) as a positive control. Secretion of active form of IL-1β was analyzed by immunoblotting. **C**, LPS-primed BMDMs were treated with the indicated dosage of GB with/without nigericin (NG), flagellin, or dsDNA. Secretion of IL-1β was analyzed by immunoblotting. **D**, BMDMs were treated with the indicated dosage of GB or LPS (10 ng/mL). Expression of *Pro-IL-1β* and *TNFα* mRNAs was measured by RT-PCR. All immunoblot and RT-PCR data shown are representative of at least three independent experiments.
